# The Role of Genetic Variation of BMI, Body Composition, and Fat Distribution for Mental Traits and Disorders: A Look-Up and Mendelian Randomization Study

**DOI:** 10.3389/fgene.2020.00373

**Published:** 2020-04-21

**Authors:** Triinu Peters, Lena Nüllig, Jochen Antel, Roaa Naaresh, Björn-Hergen Laabs, Lisa Tegeler, Chaima Amhaouach, Lars Libuda, Anke Hinney, Johannes Hebebrand

**Affiliations:** ^1^Department of Child and Adolescent Psychiatry, Psychosomatics and Psychotherapy, University Hospital Essen, University of Duisburg-Essen, Essen, Germany; ^2^Institute of Medical Biometry and Statistics, University of Lübeck, University Hospital Schleswig-Holstein, Lübeck, Germany

**Keywords:** body composition, abdominal obesity, body mass index, mental disorders, mental traits, genetic overlap, GWAS, Mendelian randomization

## Abstract

Anthropometric traits and mental disorders or traits are known to be associated clinically and to show genetic overlap. We aimed to identify genetic variants with relevance for mental disorders/traits and either (i) body mass index (or obesity), (ii) body composition, (and/or) (iii) body fat distribution. We performed a look-up analysis of 1,005 genome-wide significant SNPs for BMI, body composition, and body fat distribution in 15 mental disorders/traits. We identified 40 independent loci with one or more SNPs fulfilling our threshold significance criterion (*P* < 4.98 × 10^–5^) for the mental phenotypes. The majority of loci was associated with schizophrenia, educational attainment, and/or intelligence. Fewer associations were found for bipolar disorder, neuroticism, attention deficit/hyperactivity disorder, major depressive disorder, depressive symptoms, and well-being. Unique associations with measures of body fat distribution adjusted for BMI were identified at five loci only. To investigate the potential causality between body fat distribution and schizophrenia, we performed two-sample Mendelian randomization analyses. We found no causal effect of body fat distribution on schizophrenia and vice versa. In conclusion, we identified 40 loci which may contribute to genetic overlaps between mental disorders/traits and BMI and/or shape related phenotypes. The majority of loci identified for body composition overlapped with BMI loci, thus suggesting pleiotropic effects.

## Introduction

Genome-wide association studies (GWAS) have recently identified a large number of genome-wide significant loci with an impact on body mass index (BMI; kg/m^2^)/obesity, body composition, and body fat distribution on the one hand, and independently on mental disorders and quantitative mental traits on the other hand. The most recent GWAS for anthropometric traits were all published in 2019 substantiating the rapidly evolving dissection of their polygenic basis: (a) for BMI, 941 near-independent genome-wide significant SNPs at 536 polygenic loci have been detected (Yengo et al., 2018). (b) A GWAS for whole-body lean body mass (LBM; adjusted for sex, age, and height with or without fat mass adjustments) revealed seven LBM loci (Karasik et al., 2019). Another very recent GWAS for the proportion of body fat distribution in arms, legs, and trunk on 362,499 individuals from the UK Biobank identified 98 independent loci, 29 of which had not previously been associated with anthropometric traits including BMI (Rask-Andersen et al., 2019). (c) The most recent GWAS for body fat distribution as measured by waist-to-hip ratio adjusted for BMI (WHR_adjBMI_) reported 463 independent signals (*P* < 5 × 10^–9^) in 346 loci (Pulit et al., 2019). Because BMI and waist circumference (WC), hip circumference (HC), and WHR are more or less strongly correlated in adults (correlation coefficients of approximately 0.8 for BMI,WC, and HC, respectively; 0.3 for BMI and WHR; see e.g., Schneider, 2010), WC, HC, and WHR have been analyzed as such in GWAS (Heard-Costa et al., 2009; Wen et al., 2014; Shungin, 2015), but have also been adjusted for BMI (Shungin, 2015; Graff et al., 2017; Pulit et al., 2019).

Substantial progress has also been achieved toward the elucidation of the polygenic basis of several mental disorders [schizophrenia (Ripke, 2014), autism (ASD; Grove et al., 2017), major depressive disorder (MDD; Wray et al., 2018), bipolar disorder (BD; Stahl et al., 2017), attention deficit/hyperactivity disorder (ADHD; Li et al., 2014; Demontis et al., 2018), anxiety disorders (Otowa et al., 2016), and anorexia nervosa (AN; Duncan et al., 2017)] and quantitative mental traits [intelligence (Sniekers et al., 2017; Hill et al., 2018), educational attainment (Okbay, 2016b; Lee et al., 2018), tiredness (Deary, 2018), openness (de Moor et al., 2012), conscientiousness (de Moor et al., 2012; Lo et al., 2017), neuroticism (Okbay, 2016a; Lo et al., 2017; Luciano et al., 2018), well-being (Okbay, 2016a), and depressive symptoms (Okbay, 2016a)]. The highest number of genome-wide significant loci (*n* = 1,271) has been detected for educational attainment (Lee et al., 2018). With respect to mental disorders, the most loci (*n* = 145; Pardinas et al., 2018) have been identified for schizophrenia, for other disorders only one genome-wide significant hit [e.g., anxiety disorders (Otowa et al., 2016), AN (Duncan et al., 2017), conscientiousness (de Moor et al., 2012), and tiredness (Deary, 2018)] has been described.

To address the association of obesity with mental disorders, both clinical and population-based studies have been conducted cross-sectionally and longitudinally (Richardson et al., 2003; Patalay and Hardman, 2019). Particularly well analyzed are associations between obesity and depression (Luppino et al., 2010; Berkowitz and Fabricatore, 2011; Mühlig et al., 2015; Milaneschi et al., 2019) and ADHD (Cortese et al., 2016; Nigg et al., 2016), respectively. For obesity and MDD, a bidirectional, partly sex dependent relationship applies, entailing that obesity can precede MDD and vice versa (Luppino et al., 2014; Mühlig et al., 2015; Coryell et al., 2016). An association with obesity was also reported for BD and schizophrenia (Gurpegui et al., 2012). Because of the adverse effect of weight gain inherent to several psychotropic drugs, especially atypical antipsychotics (Almandil et al., 2013), a reliable assessment of the respective associations can only be based on drug-naïve or previously untreated patients (Gurpegui et al., 2012). A meta-analysis for BMI and WHR in never or minimally treated patients with psychosis or schizophrenia found lower BMI and elevated WHR in patients compared to healthy controls. No differences in WC were observed (Shah et al., 2019).

Observed clinical relationships between anthropometric traits BMI, obesity, body composition, and fat distribution have partially been supported by the detection of significant genetic correlations; in addition, genetic correlations have gone beyond established clinical associations. Significant negative genetic correlations have been described for BMI (or obesity) with educational attainment, intelligence, and neuroticism as well as with schizophrenia and BD. Positive genetic correlations have been found between BMI (or obesity) and ADHD and depression (Bulik-Sullivan et al., 2015; Zheng, 2017; Hill et al., 2018; Richardson et al., 2019b)^1^: (a) The negative genetic correlations between body fat percentage (BF%) and educational attainment and schizophrenia should also be highlighted (Richardson et al., 2019b)^1^; (b) At the functional level, several genes coding for proteins involved in synaptic function have been associated with substance use, obesity-related traits, responses to mental and physical stress, heart rate, and blood pressure measurements (Nikpay et al., 2012).

Thus, complex anthropometric and mental phenotypes may reveal extensive overlapping genetic factors. Research is clearly warranted to identify genetic variation involved both in the (1) regulation of body weight, body composition, and body fat distribution and (2) in mental disorders/traits. Particularly, body fat distribution is of great importance, because visceral fat (abdominal obesity) is the main factor underlying the clinical association between obesity and hypertension and type 2 diabetes mellitus (Snijder et al., 2006). Because stress is presumed to play an important role in the development of abdominal obesity (Björntorp, 2001; Geiker et al., 2018; van der Valk et al., 2018), as well as in the pathogenesis of mental disorders (Chrousos, 2009), a genetic link especially with body fat distribution may be particularly pronounced.

The purpose of the current study was to perform for the first time a systematic look-up analysis to assess the potential overlap of genome-wide significant loci for a range of anthropometric traits with mental disorders/traits. Methodologically similar to our previous study, which assessed the role of metabolite SNPs in mental disorders/traits (Hebebrand et al., 2018), we initially identified all genome-wide significant SNPs for the anthropometric traits. Look-ups were subsequently performed in GWAS data sets for 15 mental disorders/traits. For our initial look-up analysis we used data from the GWAS catalog in December 2017. We aimed for more detailed analyses in follow-up studies. Thus, to keep up with the rapid pace in the GWAS field, we have used the latest data set for each phenotype for our *post hoc* analyses [effect sizes and directions, Mendelian randomization; e.g., for BMI (Yengo et al., 2018), body fat distribution (Pulit et al., 2019) or schizophrenia (Pardinas et al., 2018), and MDD (Wray et al., 2018)].

The term “mental disorders” refers to disorders delineated in DSM-5 (American Psychiatric Association, 2013). Mental traits refer to quantitative personality/character, behavioral and socio-psychological traits. We use the overarching term mental phenotypes to refer to both mental disorders and mental traits. Apart from the genetic contribution, there are strong environmental, behavioral, and social components to some of these phenotypes (Meyer-Lindenberg and Tost, 2012).

To exemplarily investigate the potential causality of SNPs for body fat distribution in schizophrenia (and vice versa) in *post hoc* analyses, we performed a two-sample Mendelian randomization (MR) analysis with WHR_adjBMI_ as exposure and the risk for schizophrenia as outcome. In the reverse MR we used the risk of schizophrenia (Richardson et al., 2019a) as exposure and WHR_adjBMI_ as outcome.

## Materials and Methods

### Look-Ups

#### Selection of Relevant SNPs

**FIGURE 1:**
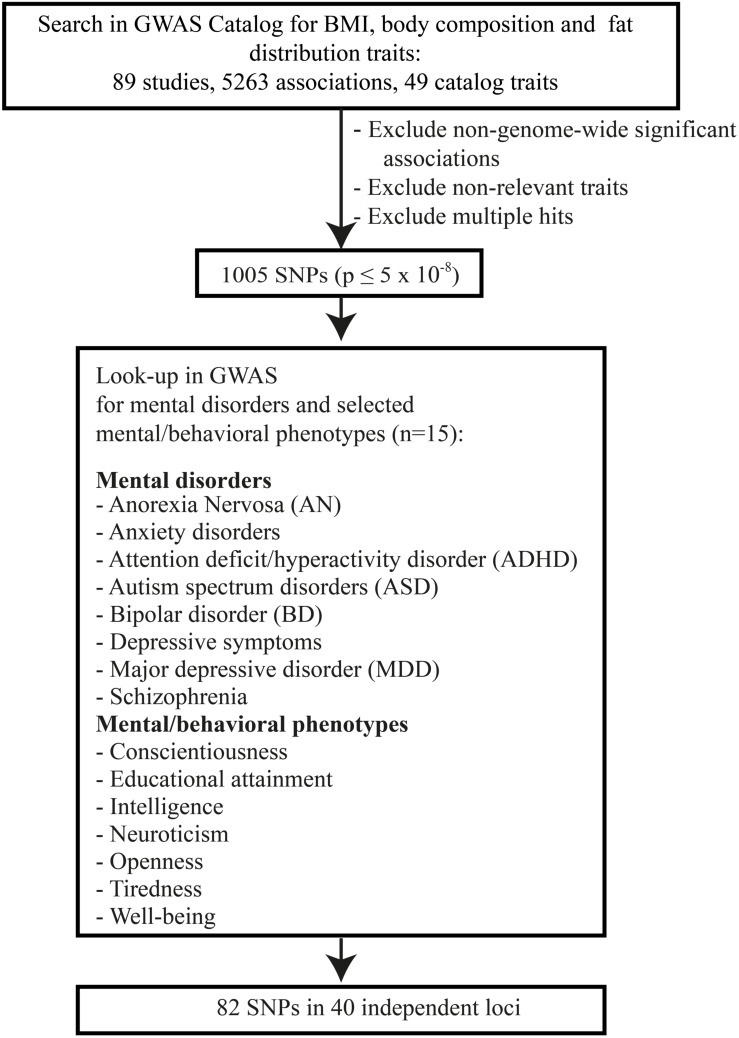
Flow chart of the conducted look-up analysis.

Genome-wide significant SNPs (*P* ≤ 5 × 10^–8^) associated with (i) BMI (obesity), (ii) body composition, and/or (iii) body fat distribution (Figure 1, search terms in Supplementary Table S1) were extracted from the GWAS Catalog version v1.0^2^ (Welter et al., 2014) on 12 December 2017. GWAS Catalog provides uniform data sets (positions, genes, and systematized information on the studies).

The identified studies were independently reviewed by three authors (LL, TP, and JH) in order to exclude anthropometric traits that did not fit the purpose of the analysis. Single different ratings were resolved by consensus. This selection process resulted in the exclusion of some traits from further analyses with a focus on a disease (other than obesity), e.g., BMI in smokers, body mass in chronic obstructive pulmonary disease, BMI (change over time) in cancer, and osteoporosis.

The look-up of the identified SNPs was performed in March 2018 in publically available GWAS data sets for mental phenotypes, taking into account only studies that had identified at least one genome-wide significant locus as previously suggested (Hebebrand et al., 2018). We added a more recent dataset for MDD because of the great importance of this disorder. Based on different consortia the following studies were included: (a) the Psychiatric Genetics Consortium (PGC)^3^: schizophrenia (Ripke, 2014), ADHD (Demontis et al., 2018), BD (Sklar, 2011), and ASD (Autism Spectrum Disorder Working Group of the Psychiatry Genomics Consortium, 2015; downloaded between 22 November 2016 and 12 February 2017), AN (Duncan et al., 2017; 26 September 2017), MDD (Wray et al., 2018; 2 May 2018); (b) the Social Science Genetic Association Consortium (SSGAC)^4^: educational attainment (Okbay, 2016b), neuroticism, well-being, and depressive symptoms (Okbay, 2016a; 22 November to 12 December 2016); (c) Anxiety Neuro Genetics Study: anxiety disorder (Otowa et al., 2016; 28 November 2016), (d) Complex Treat Genetic Lab (GTCLab): intelligence (Sniekers et al., 2017; 29 June 2017); (e) The Genetics of Personality Consortium (GPC): conscientiousness and openness (de Moor et al., 2012; 27 September 2017); (f) The Centre for Cognitive Ageing and Cognitive Epidemiology (CCACE): tiredness (Deary, 2018; 19 October 2017; Supplementary Table S3).

To define independent loci we used a distance criterion of ±500 kb surrounding the middle SNP of a chromosomal locus (Locke et al., 2015). Consecutive numbers were assigned to each locus (Supplementary Table S4; for MDD loci see Supplementary Table S5). Furthermore, linkage disequilibrium (*r*^2^) was checked within each locus and, in case of linkage equilibrium between two SNPs (*D*′ < 0.01, *r*^2^ < 0.01)^5^, these were regarded as independent loci.

Independent data sets for confirmatory look-up analyses were available for (a) educational attainment (Davies et al., 2016) [*n* = 111,114, downloaded on 4 December 2017 (Leslie et al., 2014), based on data from the CHARGE and COGENT consortia, and UK Biobank]. These study groups are independent of the 65 study groups included in the study of Okbay (2016b); and (b) neuroticism (Luciano et al., 2018; UK Biobank sample size; *n* = 329,821; 17 April 2018; Supplementary Table S3).

Genome-wide association studies for mental phenotypes and anthropometric traits have been conducted with study groups that mainly included people of European descent. However, single anthropometric studies were performed in Japanese (Okada et al., 2012; Akiyama et al., 2017), African (Monda et al., 2013; Graff et al., 2017), and East-Asian populations (Wen et al., 2014).

To provide the reader with a synopsis, Tables 1, 2 were derived from Supplementary Tables S4, S5, which illustrate the complete results of the look-ups. Tables 1, 2 depict one representative SNP for each study-wide significant locus, which in case of loci tagged by >1 SNP was identified by (1) if applicable, the highest number of mental phenotypes hits (*P* < 4.98 × 10^–5^), (2) among these SNPs the one with the lowest *P*-value was chosen.

**TABLE 1:** Results of look-up analyses for the lead anthropometric trait BMI.

Locus No.	Lead SNP	Anthropometric trait/phenotype within the locus	Chromo-somal locus	Reported genes	EdAtt	Intelligence	Schizo-phrenia	Bipolar disorder	ADHD	Depression symptoms	MDD	Neuroticism	Well-being	Effect direction^&^
1	rs3101336	BMI, obesity, WC, and HC	1p31.1	*NEGR1, LINC01360, KRT8P21*	9.7E−07	4.0E−07				1.3E−05^#^	**8.0E−08**			C: BMI↑, Obesity↑, HC↑, WC↑, IQ↑, EdAtt↑, MDD↑, and Depression↑

2	rs12401738	BMI, obesity, WC, and HC	1p31.1	*FUBP1, ZZZ3, DNAJB4*	5.2E−07									A: BMI↑, HC↑, WC↑, and EdAtt↓

3	rs1561288	BMI, obesity, HC, and WC_adjBMI_	2p23.3	*POMC*			1.4E−05							C: BMI↑, HC (n.s.), WC_adjBMI_↓*, SZP↓

4	rs7613875	BMI	3p21.31	*MON1A, RBM6*	**4.4E**−**16**									A: BMI↑ and EdAtt↓

5	rs2710323	BMI, WHR, WC_adjBMI_, HC_adjBMI_, and WHR_adjBMI_	3p21.1	*NT5DC2, PBRM1, ITIH1, NEK4, ITIH3, ITIH4*			**9.1E**−**11**	3.7E−06						T: BMI↓, WHR↑*, WC_adjBMI_↑, HC_adjBMI_ (n.s.), WHR_adjBMI_↑, SZP↑, and Bipolar↑

6	rs2325036	BMI, obesity, WC, and HC	3p12.1	*CADM2*	1.8E−07									A: BMI↑,WC↑, HC↑, and EdAtt↓

7	rs7621025	BMI and WC_adjBMI_	3q22.3	*STAG1*			9.3E−06							C: BMI↑, WC_adjBMI_ (n.s.), and SZP↓

8	rs1516725	BMI, obesity, WC, HC, and HC_adjBMI_	3q27.2	*ETV5*	1.5E−05									C: BMI↑, obesity↑, WC↑, HC↑, HC_adjBMI_ ↓*, and EdAtt↑

9	rs13107325	BMI	4q24	*SLC39A8*		1.1E−07	**1.5E**−**12**							T: BMI↑, SZP↑, and IQ↓

10	rs7720894	BMI	5q12.1	*ZSWIM6*	**5.0E**−**09**		**1.2E**−**10**							C: BMI↓, EdAtt↓, and SZP↑

11	rs1846974	BMI	5q14.3	*LINC00461, LOC645323*	6.6E−06							3.1E-06		A: BMI↑. EdAtt↑, and Nt↑

12	rs40067	BMI and WC_adjBMI_	5q21.3	*FBXL17*					1.6E−05					G: BMI↑, WC_adjBMI_ (n.s.), and ADHD↑

13	rs329120	BMI	5q31.1	*JADE2*	3.9E-07									T: BMI↓ and EdAtt↑

14	rs3800229	BMI, WC and HC	6q21	*FOXO3, FOXO3A*		**5.4E**−**10**	1.1E−07							T: BMI↑, WC↑, HC↑, IQ↑, and SZP↓

15	rs2185027	BMI	6q25.2	*RGS17*	2.9E−05									C: BMI↑ and EdAtt↓

16	rs7779181	BMI	7p14.3	*PDE1C, LOC100130673*		2.5E−06								C: BMI↑ and IQ↓

17	rs10811901	BMI	9p21.3	*LINC01239, LOC101929563*	**9.5E**−**12**									A: BMI↑ and EdAtt↑

18	rs2163188	BMI	10q21.3	*REEP3*	**2.7E**−**08**									C: BMI↑ and EdAtt↓

19	rs4409766	BMI, WC, HC, WC_adjBMI_ and HC_adjBMI_	10q24.32	*ASMT/C10orf32, CYP17A1*			**1.9E**−**11**				1.9E−05			C: BMI↑, HC↑, WC↑, WC_adjBMI_ (n.s.), WHR_adjBMI_ (n.s.), SZP↓, and MDD↓

20	rs11191560	BMI	10q24.33	*NT5C2*			**6.3E**−**16**				3.6E−05			C: BMI↑, SZP↓, and MDD↓

21	rs10838738	BMI, WC, and HC	11p11.2	*MTCH2, FNBP4*								3.0E−06		G: BMI↑, HC↑, WC↑ and Nt↓

22	rs7123876	BMI	11q13.4	*ARAP1*	3.3E−06									C: BMI↑ and EdAtt↓

23	rs1569980	BMI and WC	14q12	*PRKD1, MIR548AI*	6.4E−07									C: BMI↑, WC↑*, and EdAtt↓

24	rs17522122	BMI	14q12	*AKAP6, NPAS3*			4.7E−06							T: BMI↑ and SZP↑

25	rs1559677	BMI	15q21.1	*SEMA6D*			1.0E−05							G: BMI↑, SZP↑

26	rs2008514	BMI, obesity, BF%^§^, WC, HC and HC_adjBMI_	16p11.2	*IL27, NUPR1, ATP2A1, SH2B1, APOB48R, SULT1A2, AC138894.2, ATXN2L, SBK1 TUFM,ATP2A1-AS1 RABEP2*	**9.4E**−**09**	**1.5E**−**08**								A: BMI↑, BF↑, WC↑, HC↑, HC_adjBM__I_↑, EdAtt ↓, and IQ↓

27	rs2281727	BMI	17p13.3	*SMG6*			**3.6E**−**08**							G: BMI↑ and SZP↓

28	rs4925114	BMI and HC	17p11.2	*RAI1*			1.2E−07							G: BMI↑, HC↑, and SZP↓

29	rs12150665	BMI	17q12	*GGNBP2*		2.0E−06								C: BMI↓ and IQ↑

31	rs8092503	BMI	18q21.2	*RAB27B, DYNAP*							6.9E−06		3.7E−05	A: BMI↓, MDD↑, and WB↓

32	rs2331841	BMI, obesity, BF%^§^, BFM^£^, WC, HC, and WHR	18q21.32	*MC4R*								3.3E−05		A: BMI↑, BF%↑*, WC↑, HC↑, WHR↑, and Nt↓

33	rs427943	BMI	21q22.3	*ADARB1*							3.1E−05			C: BMI↑ and MDD↑

Other traits for body composition (BF% and BFM) and body fat distribution (WC, HC, WHR, WC_adjBMI_, HC_adjBMI_, and WHR_adjBMI_) are listed, if the lead SNP or SNPs in linkage disequilibrium also resulted in genome-wide significant (P ≤ 5 × 10^–8^) associations to the respective locus. Loci shown here represent at least one study-wise significant hit (P < 4.98 × 10^–5^; see Supplementary Tables S4, S5 for complete results) for a mental phenotype. BMI, body mass index; WC, waist circumference; HC, hip circumference; WHR, waist-to-hip ratio; BFM, body fat mass; MDD, major depressive disorder; IQ, intelligence; EdAtt, educational attainment; SZP, schizophrenia; WB, well-being; adjBMI, adjusted for BMI; *P-value between 0.05 and 4.98 × 10^–5^; n.s.: the representative SNP is not significant for this phenotype, therefore, it was not possible to determine the effect direction. ^#^Data for SNP rs7550173 is shown, which is in high LD with the lead SNP rs3101336 (r^2^ = 0.47, D′ = 0.67), ^§^ body fat percentage (measured either with bioimpedance analysis or dual energy X-ray absorptiometry), ^£^body fat mass (measured with dual-energy X-ray absorptiometry scanners); ^&^upward arrows indicate positive beta-values or odds ratios >1, downward arrows negative beta-values or odds ratios <1; P-values ≤ 5 × 10^–8^ (genome-wide significant) are shown in bold.

**TABLE 2:** BMI independent loci associated with measures of body fat distribution adjusted for BMI (WC adjusted for BMI: WC_adjBMI_; HC adjusted for BMI: HC_adjBMI_; and WHR adjusted for BMI: WHC_adjBMI_; for complete overview of the results see Supplementary Table S4).

Locus No.	Lead SNP	Anthropometric trait/phenotype within the locus	Chromosomal locus	Reported genes	EdAtt	Intelligence	Schizophrenia	Effect direction^&^
34	rs615672	WC_adjBMI_ and WHR_adjBMI_	6p21.32	*HLA-DRB1*			5.7E−07	G: WC_adjBMI_↑, WHR_adjBMI_↑, and SZP↑

35	rs11156429	WC_adjBMI_ and HC_adjBMI_	6q16.3	*HACE1*			2.3E−06	T: WC_adjBMI_↑, HC_adjBMI_↑, and SZP↑

36	rs1538170	HC_adjBMI_	6q22.32	*CENPW*		4.9E−05		T: HC_adjBMI_↑, IQ↑

37	rs1144	WC_adjBMI_	7q22.3	*SRPK2*			1.3E−06	T: WC_adjBMI_↓ and SZP↓

38	rs1727294	HC_adjBMI_	12q24.31	*PITPNM2*	**9.1E**−**14**	3.4E−05	**4.7E**−**09**	A: HC_adjBMI_↑, EdAtt↑, IQ↑, and SZP↑

39	rs4765219	WC_adjBMI_ and WHR_adjBMI_	12q24.31	*DNAH10, CCDC92*			1.8E−05	C: WC_adjBMI_↑, WHR_adj__BMI_↑, and SZP↓

40	rs181553	HC_adjBMI_	18q21.1	*DYM*			3.6E−06	A: HC_adjBMI_↑ and SZP↑

*BMI, body mass index; WC, waist circumference; HC, hip circumference; WHR, waist-to-hip ratio; MDD, major depressive disorder; IQ, intelligence; EdAtt, educational attainment; SZP, schizophrenia; WB, well-being; adjBMI, adjusted for BMI; ^&^upward arrows indicate positive beta-values or odds ratios >1, downward arrows negative beta-values or odds ratios <1; P-values >5 × 10−^8^ (genome-wide significant) are shown in bold.*

Based on supplementary files and summary statistics effect directions of the alleles for mental phenotypes and the anthropometric traits were described for each representative SNP (*P* < 4.98 × 10^–5^)^6,7^. In addition, the effect directions of the alleles were included for each anthropometric trait, for which that particular locus showed at least one hit. For this purpose, summary statistics for HC, WC, WHR, HC_adjBMI_, WC_adjBMI_, WHR_adjBMI_ (Shungin, 2015; European descent and sex-combined), BF% (Lu et al., 2016), and BMI (European descent; Locke et al., 2015; Yengo et al., 2018) were used.

If a SNP for BMI was found only in a GWAS study based on participants of non-European descent, we reviewed the effect directions and allele frequencies in GWAS of European descent (Locke et al., 2015; Yengo et al., 2018). Summary statistics were downloaded on 30 January 2018 for BMI (Locke et al., 2015) on 14 February 2019, for BMI (Yengo et al., 2018) on 26 February 2019 for WC, WHR, HC_adjBMI_, WC_adjBMI_, and WHR_adjBMI_ (Shungin, 2015); for BF% (Lu et al., 2016) on 26 February 2019^8,9^.

#### Statistical Analyses

To control for the overall type I error rate in the look-up, Bonferroni correction was applied. We assumed a univariate multiple regression model for each mental phenotype and independence of SNPs. This entailed a Bonferroni correction for 1,005 tests (1,005 SNPs), resulting in a threshold *P*-value of 4.98 × 10^–5^. We did not prune SNPs in high LD to adhere to a conservative approach. For confirmatory analyses, the same *P*-value (4.98 × 10^–5^) was considered as significant.

### Mendelian Randomization

We performed a Mendelian randomization study to exemplarily investigate the causality between body fat distribution and schizophrenia. Body fat distribution data were obtained from the latest GWAS analysis (Pulit et al., 2019; 694,649 individuals), which explained about 3.9% of the variance in WHR_adjBMI_. As data source for the risk for schizophrenia the GWAS analysis; with 40,675 cases and 64,643 controls (145 independent loci) was used (Pardinas et al., 2018). SNP-based heritability was estimated at 24% (Pardinas et al., 2018; Pettersson et al., 2019). We conducted a two-sample MR analysis with WHR as risk factor (exposure) and schizophrenia as outcome variable and the reverse analysis. In order to conduct a MR study, three core assumptions must be fulfilled (Haycock et al., 2016; König and Greco, 2018). Therefore, we conducted an *F*-test to test the weakness of the instrument (Del Greco et al., 2015; Burgess et al., 2017). We examined horizontal pleiotropy by estimating the intercept of Egger’s regression (Burgess and Thompson, 2017). To test heterogeneity of the instrument variable, we used Cochran’s Q-statistic.

We carried out MR using two statistical methods to estimate overall ratio effects: the inverse-variance weighted method (IVW) and the MR-Egger method. IVW assumes that the all ratio estimates provide independent evidence on the causal effect and there is no pleiotropic effect. So IVW assumes that all genetic variants are valid instrumental variables. There is no intercept term in the regression model (Burgess and Thompson, 2017). In MR-Egger the intercept term is estimated as part of the analyses. The intercept term can be interpreted as the average pleiotropic effect of a genetic variant included in the analyses. A non-zero intercept from MR-Egger shows that there is directional pleiotropy, or that instrumental variable assumptions are violated, or both (Burgess and Thompson, 2017). Consideration of MR-Egger estimates and intercept terms, and comparison of IVW and MR-Egger estimates is helpful to interpret results of a MR analysis (Burgess and Thompson, 2017).

We performed a power analysis to estimate whether our analysis, given sample size, proportion of cases in the study and the proportion of variance explained, is sufficient to detect a true casual effect (Brion et al., 2013). In order to investigate the relationship between study accuracy and effect size, we created a funnel plot (Haycock et al., 2016; Katikireddi et al., 2018). To examine whether an individual data point (SNP) has a large influence on the regression coefficients, we calculated the IVW regression by leaving each genetic variant out in turn (Burgess and Thompson, 2017). To visualize the results, forest and scatter plots were used. We also performed MR with Robust Adjusted Profile Score (RAPS), which estimator is more robust to pleiotropy (Zhao et al., 2019). All tests were performed using the software “R,” version 3.5.2 and R-packages for performing 2-sample MR^10^ and RAPS.^11,12^

## Results

### Look-Ups

A total of 1,005 genome-wide significant (*P* ≤ 5 × 10^–8^) SNPs from 56 studies were identified for BMI/obesity, body composition, and/or body fat distribution (Supplementary Table S2). Upon Bonferroni correction for multiple testing (*P* < 4.98 × 10^–5^), 82 SNPs at 40 genomic loci were associated with one or more mental phenotypes (Tables 1, 2; see Supplementary Tables S4, S5 for complete overview), among which the four mental phenotypes educational attainment (16 loci), intelligence (7 loci), schizophrenia (18 loci), and MDD (5 loci) figured prominently. Single loci only were detected for BD, neuroticism, ADHD, depressive symptoms, and well-being. No hits were detected for the remaining six phenotypes (ASD, AN, anxiety, tiredness, conscientiousness, and openness).

For eleven loci, significant hits (*P* < 4.98 × 10^–5^) for more than one mental phenotype – mainly encompassing educational attainment, intelligence and schizophrenia – were identified. Locus 1p31.1 (rs3101336, rs7550173, and comprising *NEGR1*) was significantly associated with the maximal number of four phenotypes (educational attainment, intelligence, depressive symptoms, and MDD).

The seven of the 40 loci that were not identified via BMI SNPs pertained to different measures of body fat distribution adjusted for BMI (WC_adjBMI_, HC_adjBMI_, and/or WHR_adjBMI_; Table 2). All but one locus were associated with schizophrenia.

We analyzed effect directions of all alleles of significant SNPs (*P* < 4.98 × 10^–5^). For the SNPs in Table 1, we looked up the effect directions for all phenotypes in respective locus (BMI, body composition, body fat distribution, and mental phenotypes) in the respective summary statistics (Locke et al., 2015; Shungin, 2015; Lu et al., 2016; Yengo et al., 2018). As expected, the effect directions were always identical for BMI, obesity, non-adjusted HC and WC, respectively.

Table 3 provides an overview of the effect directions of all detected significant (*P* < 4.98 × 10^–5^) SNP associations separated according to associations with BMI (irrespective of additional associations to other traits; for loci see Table 1) or with unique associations pertaining to BMI adjusted measures (for loci see Table 2). Opposite effect directions of alleles associated with a higher BMI were observed twice as often for educational attainment, intelligence, and schizophrenia than concordant directions.

**TABLE 3:** Overview of effect directions at the genetic loci for BMI/obesity (see Table 1).

Phenotypes	Number of loci with effect directions in the same and opposite directions	
	↑↑	↑↓
**Educational attainment**		
BMI	5	10
Measures adjusted for BMI	1	0
**Intelligence**		
BMI	2	4
Measures adjusted for BMI	2	0
**Schizophrenia**		
BMI	4	8
Measures adjusted for BMI	5	1
**Bipolar disorder**		
BMI	1	1
Measures adjusted for BMI	0	0
**ADHD**		
BMI	1	0
Measures adjusted for BMI	0	0
**Depressive symptoms**		
BMI	1	0
Measures adjusted for BMI	0	0
**MDD**		
BMI	2	3
Measures adjusted for BMI	0	0
**Neuroticism**		
BMI	1	2
Measures adjusted for BMI	0	0

*Anthropometric measures adjusted for BMI (WHR_adjBMI_, WC_adjBMI_, and HC_adjBMI_; see Table 2) and mental phenotypes; loci listed for measures adjusted for BMI refer to the total of seven loci uniquely identified via these adjusted measures. Upward arrows indicate positive beta-values or odds ratios >1, downward arrows negative beta-values or odds ratios <1. BMI, body mass index; all BMI dependent body measures.*

### Confirmatory Analyses

We performed confirmatory look-up analyses for educational attainment and neuroticism in the most recent and independent GWAS datasets for SNPs that fulfilled our *P*-value threshold of *P* < 4.98 × 10^–5^. No independent data sets were available for other mental phenotypes (Supplementary Table S3). Twenty two of the 38 SNPs for educational attainment revealed *P*-values below *P* < 4.98 × 10^–5^ (10 of 15 loci). For neuroticism, five of seven SNPs (three of four independent loci) were confirmed in an independent dataset (Supplementary Table S6).

### Mendelian Randomization

We performed a MR analysis to investigate the causal effect of body fat distribution, measured as WHR_adjBMI_, on risk of schizophrenia (for data on the 328 genome wide significant SNPs associated with WHR_adjBMI_, see Supplementary Table S9). With *F* = 85.907 the *F*-statistic indicates strong instrumental variables. After removing palindromic and ambiguous SNPs (*n* = 9), we included 319 SNPs in the analysis. The overall estimates, calculated by IVW or MR Egger, did not reveal associations between WHR_adjBMI_ and risk of schizophrenia (IVW, *b* = 0.0062, *P* = 0.9107; Supplementary Table S10 and Supplementary Figures S4–S6), although in the single SNP analyses, 47 SNPs showed a significant result (Supplementary Table S11). Sensitivity analyses using the leave-one-out approach confirmed the lack of associations (Supplementary Figure S7). There was no evidence for pleiotropy (MR-Egger intercept = −0.0003194; *P* = 0.8991). The heterogeneity test was significant [*Q*(*df* = 317) = 860.0176, *P* = 1.12 × 10^–51^]. Power analysis revealed that our MR analyses had an 80% power to detect an odds ratio (OR) of 1.09 and 100% power to detect an OR of 1.28 for schizophrenia (Supplementary Figure S8). The more robust method RAPS also showed no causal effect of body fat distribution on schizophrenia [RAPS main function: beta(hat) = 0.0059, SE = 0.03291, *P* = 0.858; RAPS overdispersed function: beta(hat) = −0.0744, SE = 0.1115, *P* = 0.504].

To test reverse causality, we performed an MR analysis where we considered the risk of schizophrenia as a risk factor (for information on the association of 142 SNPs, see Supplementary Table S12) and WHR_adjBMI_ as an outcome. After removing palindromic and ambiguous SNPs (*n* = 4), we included 138 SNPs in the analysis. *F*-test revealed that the instruments were strong (*F* = 233.896). The heterogeneity test was significant [*Q*(*df* = 136) = 771.8842, *P* = 1.60 × 10^–89^], as was the test for directional horizontal pleiotropy (MR-Egger intercept = 0.00539, *P* = 0.0077). Consequently, we must assume pleiotropy, so that further results must be assumed to be biased. Single SNP analyses revealed that 46 SNPs showed a significant causal effect on WHR_adjBMI_ with opposite effect directions The overall MR analysis with the Egger-method revealed a causal effect of schizophrenia on WHR_adjBMI_ (*b* = −0.073, *P* = 0.0098), the IVW-method, however, showed no significant effect (*P* = 0.9296; Supplementary Tables S13, S14 and Supplementary Figures S9–S11). RAPS method showed no effect [main function: beta(hat) = 0.0007, SE = 0.0025, *P* = 0.790; overdispersed function: beta(hat) = −0.0055, SE = 0.0076, *P* = 0.473].

## Discussion

We identified 82 SNPs at 40 independent loci for (i) BMI (including obesity), (ii) body composition (BFM and BF%), and/or (iii) body fat distribution (WC, HC, and WHR; adjusted and unadjusted for BMI) concomitantly associated with mental phenotypes. The majority of locus associations were detected with educational attainment (*n* = 16), intelligence (*n* = 7), schizophrenia (*n* = 18), and MDD (*n* = 5). The fact that we found only five loci (1p31.1, 10q24.32, 10q24.33, 18q21.2, and 21q22.3) common for MDD could be due to the relatively low heritability, phenotypic heterogeneity, and multifactorial etiopathogenesis of this disorder (Levinson et al., 2014; Mullins and Lewis, 2017; Milaneschi et al., 2019).

The potential relevance of the identified loci seems to be substantiated by the fact that (a) 15 SNPs loci (seven for schizophrenia, six for educational attainment, and two for intelligence) at 12 loci met the genome-wide significance threshold and (b) 11 of 18 loci were confirmed for intelligence and neuroticism in independent data sets. Nevertheless, future GWAS and meta-analyses will need to show that our significant hits with *P*-values between *P* < 4.98 × 10^–5^ and 5 × 10^–8^ indeed represent true findings for the respective mental phenotypes.

Thirty-three loci associated with mental phenotypes were identified via BMI/obesity SNPs (Table 1); for 16 of these loci, the same or additional SNPs identified via other anthropometric traits were co-localized. Thus, as expected based on high correlations between the anthropometric traits (Schneider, 2010) the SNPs for BMI unadjusted traits of body composition and body fat distribution (WC, HC, WHR, BF%, and BFM) associated with mental phenotypes were always co-associated with the loci identified via BMI SNPs. Only seven loci were unique for BMI adjusted measures of body fat distribution (Table 2). Because of the co-occurrence of nine loci identified both via BMI and measures of body fat distribution adjusted for BMI, we looked up the lead SNPs of these seven loci (Table 2) in the most recent GWAS meta-analyses for BMI (Yengo et al., 2018; Supplementary Table S7); interestingly, all but one locus (locus no. 38: rs1727294) revealed a *P*-value <0.05, one of which was even genome-wide significant (locus no. 39: rs12317176). Clearly, the overall frequent co-identification of loci for BMI and BMI adjusted measures of body fat distribution indicates an overlap between BMI and BMI adjusted body fat distribution loci with respect to the respective mental phenotypes.

Recently, Kaur et al. (2018) conducted look-up analyses in several somatic and mental disorders using genome wide significant WHR_adjBMI_ SNPs as measurement for body fat distribution/abdominal obesity. In accordance with our results, they found that two WHR_adjBMI_ variants in 3p21.1 (see locus 5 in Table 1) are associated with schizophrenia (*PBMI1* and *ITIH1*) and BD (*ITIH1*) (Kaur et al., 2018); our results indicate that BMI, HC_adjBMI_, and WC_adjBMI_, too, are associated with these mental disorders. Additionally, they reported that *LOC105375015* (rs2596500) was associated with WHR_adjBMI_ and schizophrenia, a finding we could not confirm as our analyses did not include proxy SNPs.

There are numerous indications that the clinical association between abdominal obesity and cardiometabolic risk is largely independent of BMI (Després, 2006; van Vliet-Ostaptchouk et al., 2013). For the latest genetic study on body fat distribution (WHR_adjBMI_; Pulit et al., 2019), the potential for collider bias resulting from conditioning WHR on BMI was analyzed. They found that most WHR_adjBMI_ SNPs (311 of 346) are associated with stronger standard deviation effect sizes for WHR than for BMI. Each additional (weighted) WHR_adjBMI_-raising allele was associated with a higher WHR and with a lower BMI (Pulit et al., 2019). We looked up the 346 SNPs associated with WHR_adjBMI_ (Pulit et al., 2019) in the recent GWAS for BMI (Yengo et al., 2018): 263 SNPs overlapped between the two data sets (Yengo et al., 2018; Pulit et al., 2019), 20 of which were genome-wide significant for both WHR_adjBM__I_ and BMI. In light of the high number of SNPs identified in our study that have an effect on both BMI and WHR_adjBMI_ and the less than 10% overlap (20/263) of BMI and WHR_adjBMI_ (Yengo et al., 2018; Pulit et al., 2019). SNPs we postulate that loci having independent effects on both anthropometric traits have a higher probability of playing a role in mental phenotypes.

Our data indicate that the associations between anthropometric traits and mental disorders/traits are heterogeneous as some effects were directionally consistent and for others the risk alleles pointed to opposite directions (Table 3). As expected, effect directions for BMI, obesity, HC, and WC were consistent for all the respective 16 loci; for the two loci (nos. 5 and 32; Table 1) co-identified by WHR one effect directions was concordant, the other discordant to BMI. The overall associations between genetic risks for different diseases or genetic proportion of variance across all SNPs are expressed with genetic correlations or correlations between genetic risk scores. Small to medium, but nevertheless significant genetic correlations have been detected between BMI and schizophrenia (*r*_*g*_ = −0.10, *P* = 1.7 × 10^–4^) (Bulik-Sullivan et al., 2015), BMI and ADHD (*r*_*g*_ = 0.26, *P* = 3.0 × 10^–3^), BMI and childhood intelligence (*r*_*g*_ = −0.17, *P* = 2.3 × 0^–3^) (Bulik-Sullivan et al., 2015), BMI and intelligence (*r*_*g*_ = −0.16, *P* = 1.4 × 10^–16^) (Hill et al., 2018), BMI and educational attainment (*r*_*g*_ = −0.28, *P* = 6.6 × 10^–16^) (Bulik-Sullivan et al., 2015), and BMI and AN (*r*_*g*_ = −0.18, *P* = 3.17 × 10^–7^) (Bulik-Sullivan et al., 2015; Hill et al., 2018). Between WC or WHR and educational attainment genetic correlations of −0.29 (*P* = 2.31 × 10^–14^) and −0.33 (*P* = 1.34 × 10^–18^) were reported (Bulik-Sullivan et al., 2015). The correlations between genetic risk scores for both schizophrenia and educational attainment with BF% were negative (*P* < 0.05; Richardson et al., 2019a). We were unable to find studies providing genetic correlations between BMI, body composition, and body fat distribution traits.

For schizophrenia, we identified the strongest overlap with body fat distribution traits adjusted for BMI in our look-up analysis (Table 2). Most clinical studies (Sugawara et al., 2012; Konarzewska et al., 2014; Strassnig et al., 2017), but not all (Kim et al., 2017) have found a higher BF%, BFM, visceral adipose tissue mass in normal weight patients with schizophrenia as compared to healthy controls. These clinical observational studies either considered antipsychotic medication as a covariable (Strassnig et al., 2017), discuss it as a justification for observed differences (Kim et al., 2017), or mention the inclusion of patients on antipsychotics as a limitation (Sugawara et al., 2012). Besides general limitations of observational studies such as residual confounding, directions of potential causal links between body fat distribution and schizophrenia remain unclear. MR assumes that genetic variants are not confounded by behavioral, socioeconomic, and physiological factors and genetic variants will not be influenced by the onset of disease (Davey Smith and Ebrahim, 2005). Therefore, MR can be regarded as a randomized controlled trial provided by nature. For this reason, we applied MR analysis to the most recent data to test whether there is an overall causal association between BMI adjusted body fat distribution and schizophrenia.

The MR analysis did not reveal evidence for an overarching effect of adjusted body fat distribution on the risk for this mental disorder. The power analysis revealed that our MR had a 100% power to detect an OR of 1.28 for schizophrenia. This OR is similar as those observed for single SNPs calculated in GWAS: e.g., the T-allele of rs3130820 (the SNP with the highest effect in GWAS by Pardinas et al., 2018) increases the risk of getting a diagnosis of schizophrenia by 1.281 times (SE = 0.018, *P* = 2.12 × 10^–44^); the OR of the T-allele at rs12416331 is 1.157 (SE = 0.017, *P* = 7.09 × 10^–18^). Thus, the power of our MR was sufficient to detect a clinically relevant risk for schizophrenia. In the reverse analyses about the causal effect of schizophrenia on body fat distribution, the non-zero intercept from MR-Egger indicates that there is the directional pleiotropy or the assumptions for instrumental variable are violated, or both. The Egger method showed a significant effect, but the IVW method showed none. The RAPS method, which is more robust to pleiotropy, showed no effect. Our MR analyses suggest that there is no causal effect of schizophrenia on body fat distribution.

Our study now shows that specific genetic effects at individual loci may well play a role in associations between both BMI adjusted body fat distribution and schizophrenia.

In the following, we provide the reader with an insight into potential functional implications of five of the 40 loci which we deem of particular interest^13,14,15,16,17,18^^:^

(1) Locus 1p31.1 (rs3101336, rs7550173, *NEGR1;* locus 1 in Table 1) was significantly associated with both MDD and depressive symptoms, educational attainment, and intelligence. *NEGR1* (neuronal growth regulator 1 gene), a well-known obesity-associated gene (Willer, 2009), is associated with white matter integrity (Dennis et al., 2014), and shows high expression in several brain tissues (highest median expression in frontal cortex, Supplementary Figure S1). *NEGR1* is a member of the immunoglobulin domain-containing glycosylphosphatidylinositol (GPI)-anchored neural cell adhesion molecules (IgLON; Karis et al., 2018) and involved in neural cell communication and synapse formation (Joo et al., 2019). Recently, *NEGR1* was also found to play a role in intracellular cholesterol trafficking (Joo et al., 2019). Overall, *NEGR1* seems to play a pleiotropic role due to its involvement in synaptogenesis, neuron maturation, and neurite outgrowth, which all represent essential mechanisms for sustaining brain function and development (Pischedda and Piccoli, 2015). Significantly, elevated levels of *NEGR1* transcripts were found in the dorsolateral prefrontal cortex of patients with schizophrenia compared to healthy controls (Karis et al., 2018).
(2) *CYP17A1* (cytochrome P450 family 17 subfamily a member 1 gene) in 10q24.33 (locus 19 in Table 1) is one of the well-known genes associated with adult hypertension (Levy et al., 2009). We found an association with BMI, WC, HC, WC_adjBMI_, HC_adjBMI_, and both schizophrenia and MDD. *CYP17A1* has previously been found to be associated with schizophrenia (Ripke, 2014); our detected association is novel for MDD. In the recently published multivariate study on the wellbeing spectrum, *CYP17A1* was associated with depressive symptoms and neuroticism (Baselmans et al., 2019). A potentially overarching functional implication of this locus in both somatic and mental disorders is supported (Van Woudenberg et al., 2015) by the fact that in adolescent males but not females, a *CYP17A1* SNP was associated with enhanced blood pressure reactivity to mental stress (Van Woudenberg et al., 2015). *CYP17A1* is predominantly expressed in adrenal glands (Supplementary Figure S2) and is a bi-functional key regulatory enzyme in the steroidogenic pathway with 17α-hydroxylase and 17,20-lyase activity (Yoshimoto and Auchus, 2015). *CYP17A1* is essential for the formation of all endogenous androgens (Peng et al., 2019) and many glucocorticoids (Kmetova Sivonova et al., 2017). Key substrates are pregnenolone and progesterone, which are transformed to the androgen precursor’s dehydroepiandrosterone and androstenedione (Kim et al., 2016). Several findings indicate a sex-related susceptibility for schizophrenia with males at higher risk, e.g., earlier age of onset compared to a lower susceptibility for women due to a potentially neuroprotective role of estrogens (Seeman, 1996) via effects on dopamine (DA) neurotransmission (Di Paolo, 1994; Sanchez et al., 2010; Frau et al., 2014; Godar and Bortolato, 2014). It is hypothesized that androgenic metabolites of testosterone, especially dihydrotestosterone (DHT) and androstenedione, may facilitate the development of schizophrenia-related symptoms. Quantitative real-time PCR in post-mortem brain slices of MDD patients showed a significant decrease in mRNA levels of CYP17A1 (Qi et al., 2018) and genetic variants of the rs743572 polymorphism of CYP17A1 were correlated with the intensity of depressive symptoms in post-menopausal women (Loja-Chango et al., 2016), altogether suggesting a role of CYP17A1 in mood disorders.
(3) At the locus 16p.11.2 (see locus 26 in Table 1) higher BMI, BF%, WC, HC, HC_adjBMI_, and higher risk for obesity were negatively associated with intelligence and educational attainment in line with previous evidence (Jacquemont et al., 2011). Genetic variations [SNPs, copy number variants (CNVs), and larger deletions/insertions] at 16p.11.2 have been shown to be associated with developmental delay, ASD, ADHD, schizophrenia, BD, epilepsy, intracranial volume, brain development, congenital anomalies, altered satiety response, energy imbalance, underweight, and morbid obesity (McCarthy et al., 2009; Bochukova et al., 2010; Fernandez et al., 2010; Walters et al., 2010; Jacquemont et al., 2011; Volckmar et al., 2012; Zufferey et al., 2012; Egger et al., 2014; Qureshi et al., 2014; Volckmar et al., 2015; Maillard et al., 2016; Giuranna et al., 2018; Martin-Brevet et al., 2018; Sonderby et al., 2018; Niarchou et al., 2019). The consequences of 16p11.2 CNV and larger deletions and duplications on health are broad (Crawford et al., 2019; Niarchou et al., 2019). Because the CNVs are rare (Stefansson et al., 2014), large studies and complex analytical methods are needed to detect them in GWAS (Nothen et al., 2019).
  The SNPs we found in 16p11.2 are assigned to genes *IL27, NUPR1, ATXN2L, SH2B1, TUFM, SBK1, SULT1A2, ATP2A1, APOB48R, AC138894.2, ATP2A1-AS1*, and *RABEP2* (see Supplementary Table S6); however, a total of 41 genes may be affected by the CNV in this region (Stefansson et al., 2014). The protein encoded by *IL27 (Interleukin 27)* is one of the subunits of a heterodimeric cytokine complex and is in many aspects closely tied to the cytokines IL-12 and IL-23. IL-27 may play a role in the rapid initiation of a response to an inflammatory challenge (Pflanz et al., 2002). Neuro-inflammatory processes as a result of stress-induced immune system activation (Halaris, 2019) are discussed as potential mechanisms in the pathogenesis of mental disorders, e.g., depression (Berk et al., 2013). This hypothesis was recently supported by systems genomics studies (Sharma, 2016) and clinical intervention studies with COX-2 inhibitors and aspirin (Muller, 2019). A negative phenotypical correlation of the level of systemic inflammation biomarkers with general intelligence in children also has been observed (Lee et al., 2016). Inflammation and metabolic risk factors might increase the risk of psychosis in some people (Perry et al., 2019), inflammatory mechanisms may also play a role in the pathophysiology of stress-related and anxiety disorders (Lasselin et al., 2016).
(4) In 3p12.1 we found an association between BMI (obesity) and educational attainment (rs2325036, *CADM2*; see locus 6 in Table 1). Cell adhesion molecule 2 (*CADM2*) belongs to the immunoglobulin superfamily and interacts with other cytoskeletal proteins (Biederer, 2006). It is predominantly expressed in the central and peripheral nervous systems (Pellissier et al., 2007; Morris et al., 2019; Supplementary Figure S3) and regulates, e.g., synapse formation (Rathjen et al., 2017). Obesity risk variants (Yan et al., 2018; Morris et al., 2019) were associated with increased *CADM1* and *CADM2* expression in the hypothalamus with a complex interplay of *CADM1* and *CADM2* (Rathjen et al., 2017). Reduced expression promoted a negative energy balance and weight loss in mice (Rathjen et al., 2017). The expression of both *CADM1* and *CADM2* in several neuronal circuits suggests an intertwining of body weight maintenance with motor control, but also behavioral aspects, cognition, and memory (Rathjen et al., 2017). In addition to associations with obesity risk genes (SNP rs13078807 located in the second intron of *CADM2*) novel associations of *CADM2* genetic variants with systolic blood pressure (SBP), CRP levels (Morris et al., 2019), as well as with diverse psychological traits (neuroticism, mood instability, and risk-taking) have been detected. Interestingly, other loci identified by SNPs for BMI and/or body fat distribution in the current study are involved in the immune system, too (Supplementary Table S8): *ITIH4* (Murakami et al., 2015; locus 5, 3p21.1; BP, schizophrenia), *SLC39A8* (Liu et al., 2013b; locus 9, 4q24; schizophrenia, intelligence), and *HLA-DRB1* (Arango et al., 2017; locus 34, 6p21.32; schizophrenia).
(5) We for the first time report an association of *MC4R* (locus 32; Table 1 and Supplementary Table S4) with neuroticism. *MC4R* is well known for its role in BMI/obesity (Hinney et al., 2013). Evidence from animal models links the *MC4R* to eating behavior in stress-induced anxiety-like or depression-like behavior, anhedonia, and corticosterone secretion (Lim et al., 2012; Liu et al., 2013a; Sabban et al., 2015). *MC4R* appears to interact with the *SAPAP3* gene regulating compulsive behavior and body weight (Xu et al., 2013). A higher rate of anxiety and compulsiveness in patients with AN might be mediated by the *MC4R* (Xu et al., 2013). The *MC4R* (rs17782313-C) has also been associated with elevated scores for emotional eating and craving for food (Yilmaz et al., 2015). Gain of function variants in *MC4R*, which have a weight lowering effect (Wang et al., 2010), have been associated with binge eating disorder (BED) in overweight subjects. Apart from polymorphisms exerting weak effects on BMI 166 *MC4R* mutations mostly entailing a loss in function have been identified up to date mainly in obese individuals (Hinney et al., 2013). Apart from obesity, mutation carriers showed increased lean mass, increased linear growth, hyperphagia, hyperinsulinemia, and ADHD (Loos et al., 2008; Albayrak et al., 2011; Hinney et al., 2013).

## Limitations

Limitations of our study include: (i) we conducted a look-up analysis using summary statistics, so that we could not analyze covariates. (ii) The number of hits was higher for mental phenotypes with higher heritability, higher number of genome-wide significant loci, and/or higher numbers of subjects in the respective GWAS. Therefore, we suspect a type 2 error for phenotypes with a currently low number of identified loci. (iii) We had performed a Bonferroni correction for the 1,005 SNPs. As a drawback true associations may have been missed. On the other hand, we did not correct for the number of analyzed traits. (iv) We were unable to systematically consider the ancestry/ethnicity of the study groups comprising the diverse GWAS. However, as most data were derived from individuals of European descent this should represent a minor issue only. For those loci, which were detected in non-European study groups, we checked *P*-values and effect directions in recent GWAS conducted in individuals of European descent. (v) The progress in GWAS research leads to the release of many new analyses every year. For most phenotypes the selected GWAS data sets are the most recent ones; it was, however, not possible to include the most recent analyses, e.g., for WHR_adjBMI_ (Pulit et al., 2019), BMI (Yengo et al., 2018), and schizophrenia (Pardinas et al., 2018) in our look-up analyses. We were, however, able to use these most recent studies for MR-analysis and additional look-ups. (vi) Sex- and age-stratified analyses were not possible as the respective data sets were not available for all traits despite evidence for differences in genetic regulation for BMI and body fat distribution depending on age and sex (van Vliet-Ostaptchouk et al., 2013; Winkler et al., 2015; Pulit et al., 2019; Rask-Andersen et al., 2019). For body shape (WHR_adjBMI_), age effects were not found (Winkler et al., 2015). (viii) It needs to be noted that across the literature for body fat distribution the term “adjusted for BMI” is not defined uniformly. It can either mean that the measurement (WC, HC, and WHR) is directly adjusted for BMI or that the estimation of the SNP effects in a GWAS was adjusted for BMI. For future GWAS a clear definition of the term “adjusted for BMI” is needed. (viii) We defined loci mainly via a central SNP and a surrounding region (±500 kb). However, some loci might actually represent more than one independent locus. We attempted to detect independent hits by analysis of linkage disequilibrium; nevertheless, independent SNPs within a given locus may have escaped detection. (ix) A clinically relevant aspect of the relationship between BMI and mental disorders is the susceptibility to weight gain as a side effect of treatment with especially atypical antipsychotic drugs. Because the GWAS data for the anthropometric traits are mostly based on “healthy” individuals, large scale confounding via use of psychotropic drugs *a priori* appears unlikely. (x) Study samples for some of the examined phenotypes are not completely independent. Sample overlap can potentially affect the results of cross-trait analysis. Since our analyses were conducted using summary statistics it is not possible to determine an exact number of overlapping participants between the underlying GWAS used in our analyses. To assess whether the overlap can be assumed, we looked at whether GWAS studies included in our analysis used data from the same study groups.

The GWAS study on body fat distribution (Pulit et al., 2019), which we used for our MR analysis included a total of 29 study groups of the PGC and of UK Biobank. Four cohorts (B58C-WTCCC, TWINSUK, EGCUT, and KORA) are also mentioned as sources of controls for GWAS for schizophrenia (Pardinas et al., 2018), which we used as outcome phenotype. For cases with schizophrenia CLOZUK1, CLOZUK2, and CardiffCOGS cohorts and the PGC cohorts were used. These study groups were apparently not used for the GWAS on body fat distribution (Pulit et al., 2019).

Two-sample MR assumes independent samples. Violation of this assumption will result in increased Type 1 error and biased effect estimates (Burgess et al., 2016). For a continuous outcome, bias due to sample overlap is a linear function of the proportion of overlap between the samples with IVW method. Bias due to sample overlap may be more serious for the MR-Egger method. In a simulation analyses for binary outcome with sample overlap in the controls only – as it is the case in our study – there was no detectable bias in the IV estimates even with extremely weak instruments, nor was there any inflation of type 1 error rates (Burgess et al., 2016). Accordingly, in our MR study, the sample overlap between controls of schizophrenia GWAS and study participants for GWAS on risk variable (i.e., WHR adjusted for BMI) do not lead to a bias for estimated causality in two sample MRs with binary outcome (Burgess et al., 2016).

Sample overlap can also influence the results of the look-up analysis. As an example we compared studies that were included in the GWAS on BMI (158 studies in total; Locke et al., 2015) and studies that were included in the GWAS on educational attainment (63 studies in total; Okbay, 2016b). This comparison revealed that nineteen studies were the same.

We also checked the overlapping of the study groups included in the GWAS on BMI (GIANT; Locke et al., 2015) and in the GWAS on schizophrenia (PGC; Ripke, 2014). It is possible that a certain number of controls for the GWAS on schizophrenia were the same individuals included in GWAS on BMI (overlapping cohorts were EGCUT, MIGen, WTCCC, KORA, HRN, MGS, and deCODE).

The sample overlap between the GWAS on psychiatric disorders due to comorbidity should also be considered. This topic had previously been investigated using data from PGC (LeBlanc et al., 2018). They found that the overlap was less than 2% for eight psychiatric disorders. Data sets in the study were the same as in our study for four disorders (ADHD, AN, MDD, and schizophrenia). Nevertheless, we can probably assume that the sample overlap is of a similar magnitude for studies included in our analyses.

## Conclusion

We identified several loci that were associated with BMI, body composition and/or body fat distribution, and mental disorders/traits. The SNPs for body composition and body fat distribution (WC, HC, WHR, BF%, and BFM) associated with mental disorders/traits were all simultaneously associated with BMI. Hence, an isolated effect of body fat distribution on mental phenotypes implied by clinical and epidemiological studies (Luppino et al., 2010; Berkowitz and Fabricatore, 2011; Gurpegui et al., 2012; Luppino et al., 2014; Mühlig et al., 2015; Cortese et al., 2016; Coryell et al., 2016; Nigg et al., 2016), was not substantiated. In the MR analysis, we did not detect a general causal effect of body fat distribution on schizophrenia. Our study has confirmed some known cross-phenotype associations (Brainstorm et al., 2018) but has also given some new clues for genetic loci involved in both anthropometric traits and mental phenotypes.

## Data Availability Statement

The datasets for this article are publicly available (see section “Materials and Methods”).

## Author Contributions

All authors contributed to the writing of the manuscript and approved of the final version. TP planned and supervised the project, performed the analyses, discussed the results and wrote the manuscript; LN performed the analyses and drafted the first version of the manuscript; B-HL advised and reviewed the MR analysis; AH interpreted the results and was part of the writing team; JA discussed the results and was part of the writing team; LT contributed to analyses; RN performed the MR analyses; CA provided critical input on early versions of the manuscript; LL discussed the results and commented on the manuscript; JH planned and supervised the project, discussed the results, and wrote/finalized the manuscript.

## Conflict of Interest

The authors declare that the research was conducted in the absence of any commercial or financial relationships that could be construed as a potential conflict of interest.

## Footnotes

1 http://mrcieu.mrsoftware.org/PRS_atlas/

2 http://www.ebi.ac.uk/gwas/

3 https://www.med.unc.edu/pgc/

4 https://www.thessgac.org/

5 https://ldlink.nci.nih.gov/(Machiela and Chanock, 2015)

6 https://www.snpedia.com/index.php/SNPedia

7 https://www.ncbi.nlm.nih.gov/snp

8 https://portals.broadinstitute.org/collaboration/giant/index.php/GIANT_consortium_data_files

9 ftp://ftp.ebi.ac.uk/pub/databases/gwas/summary_statistics/LuY_26833246_GCST003435

10 https://github.com/MRCIEU/TwoSampleMR

11 https://cran.r-project.org/web/packages/mr.raps/index.html

12 https://github.com/

13 www.genecards.org

14 www.ncbi.nlm.nih.gov/gene

15 www.uniprot.org (The UniProt Consortium, 2017)

16 www.omim.org http://ldsc.broadinstitute.org/ldhub/

17 https://gtexportal.org/home/(Consortium et al., 2017)

18 https://gene.sfari.org/
