# Supplementary material for: The Role of Genetic Variation of BMI, Body Composition, and Fat Distribution for Mental Traits and Disorders: A Look-Up and Mendelian Randomization Study
Source: Front Genet. 2020 Apr 21;11:373. doi: 10.3389/fgene.2020.00373 (PMC7186862; doi:10.3389/fgene.2020.00373)

**Figure S1. Expression of NEGR1**

**https://gtexportal.org/home/**

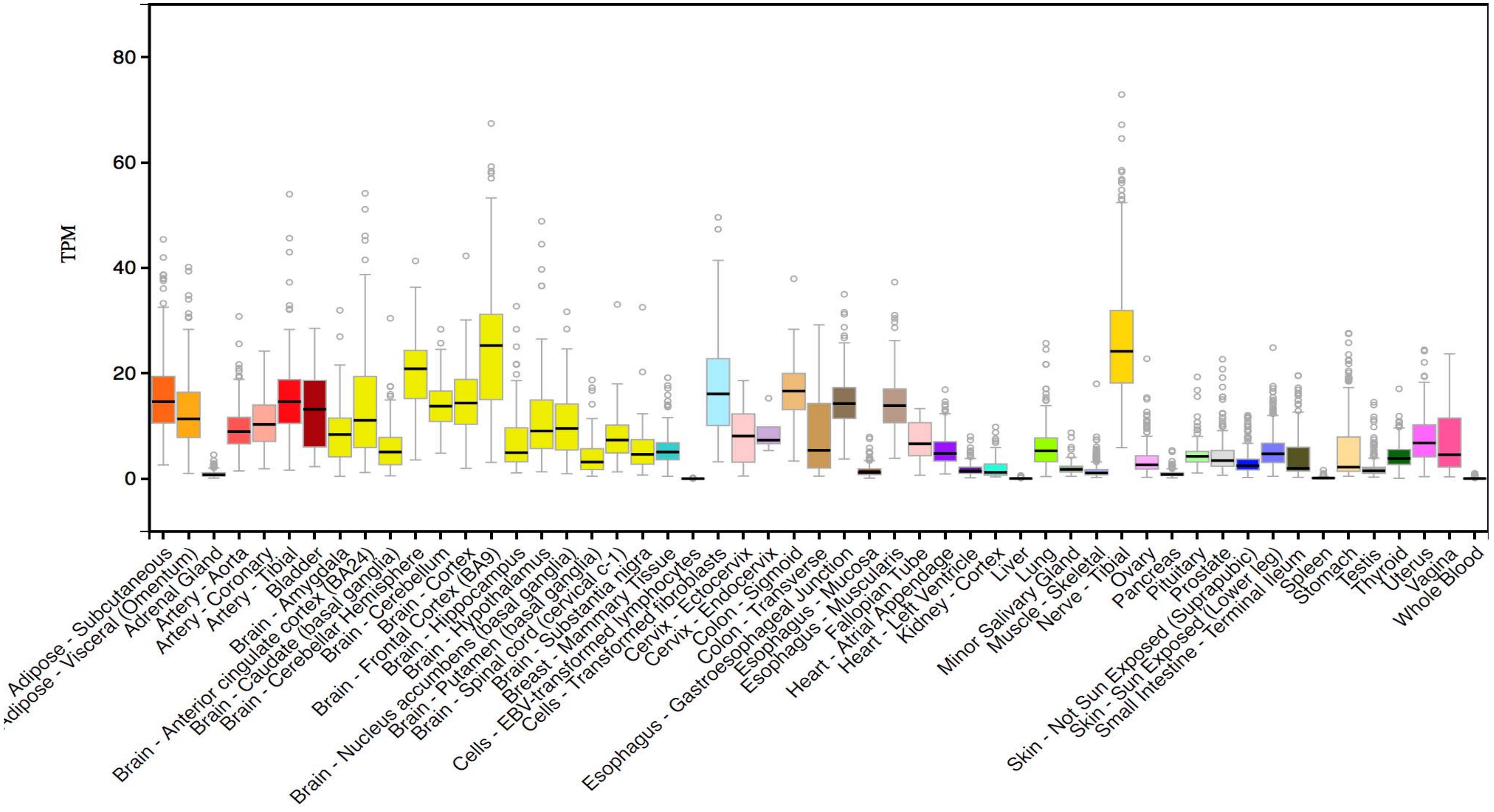

**Figure S2. Expression of CYP17A1**

**https://gtexportal.org/home/GTEx**: median Transcripts Per Million (TPM)=7642

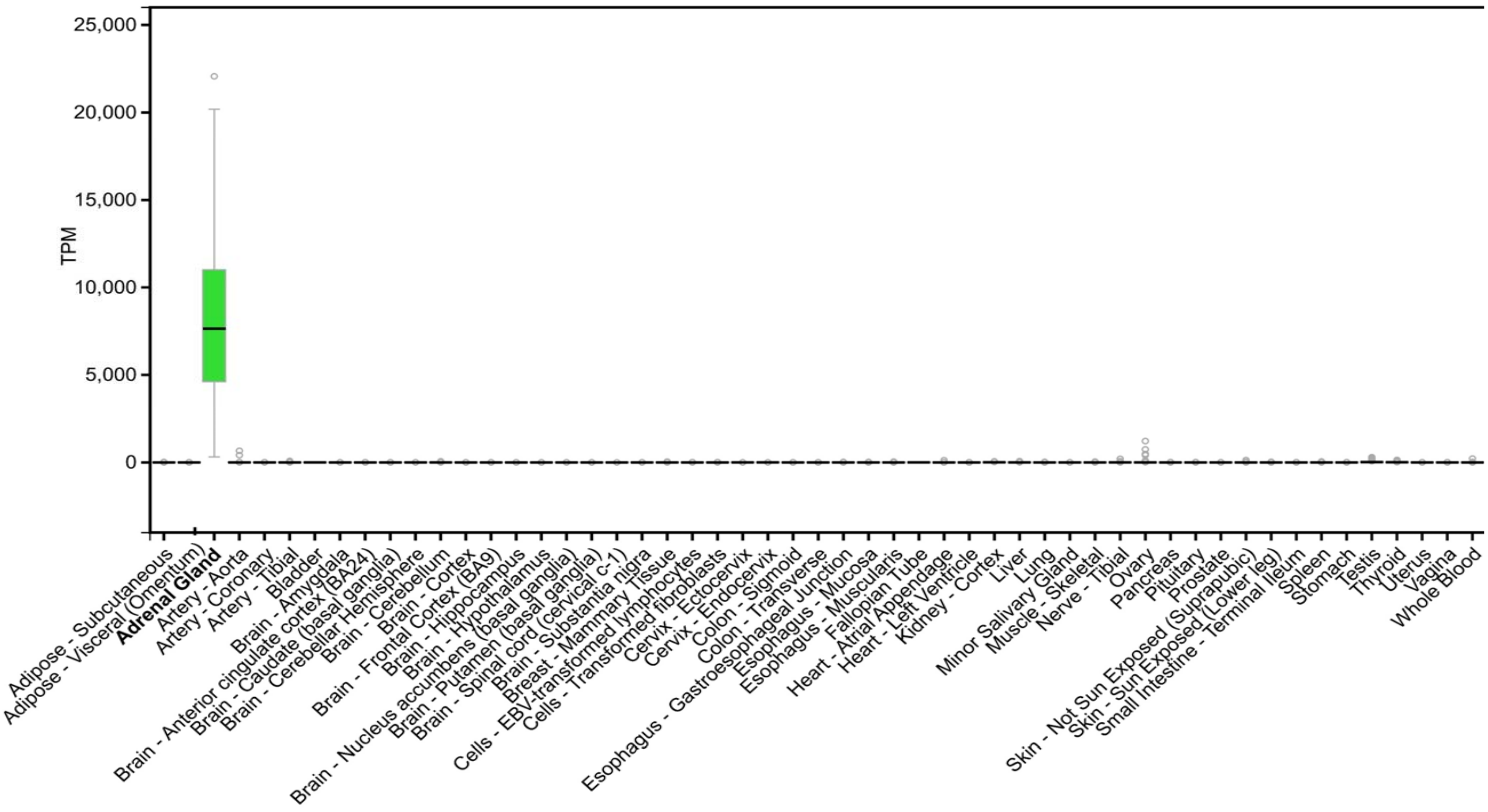

**Figure S3. Expression of CADM2**
**https://gtexportal.org/home/**

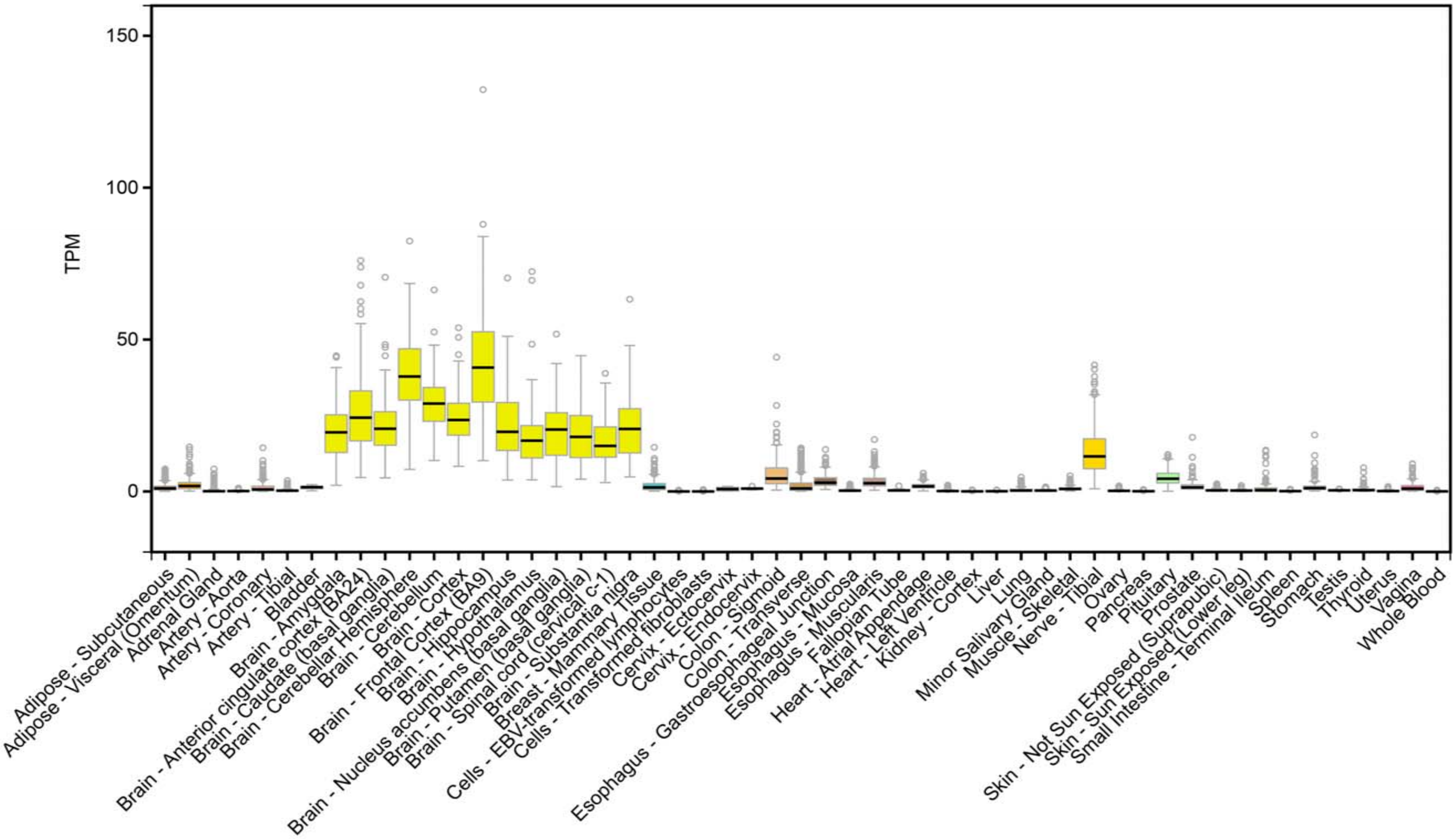

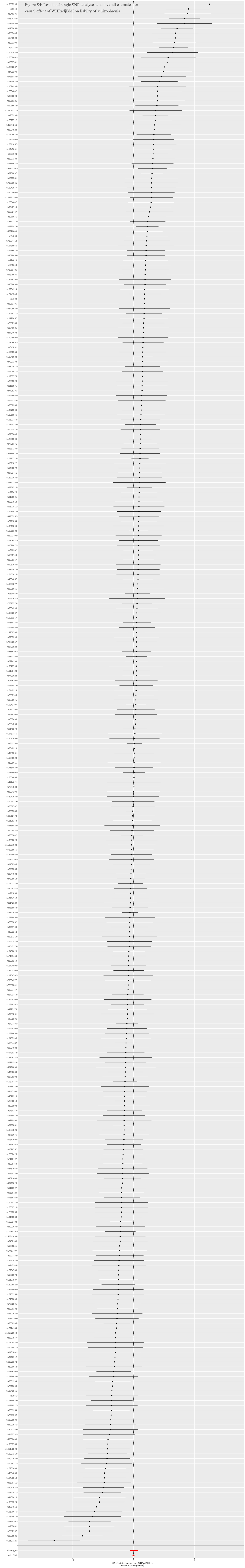


Figure S4: Results of single SNP analyses and overall estimates for causal effect of WHRadjBMI on liabilty of schizophrenia

**Figure S5. Results of the single and multi SNP MR-analyses on the association between $WHR_{adjBMI}$ and schizophrenia**

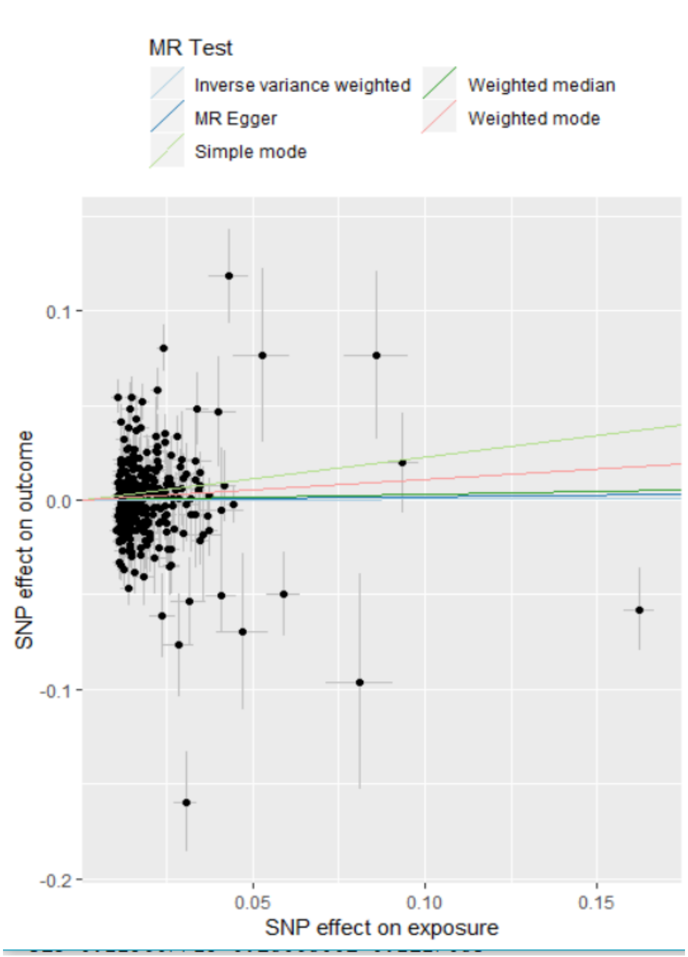


**Figure S6. Funnel plot for MR with $WHR_{adjBMI}$ as exposure and schizophrenia as outcome**

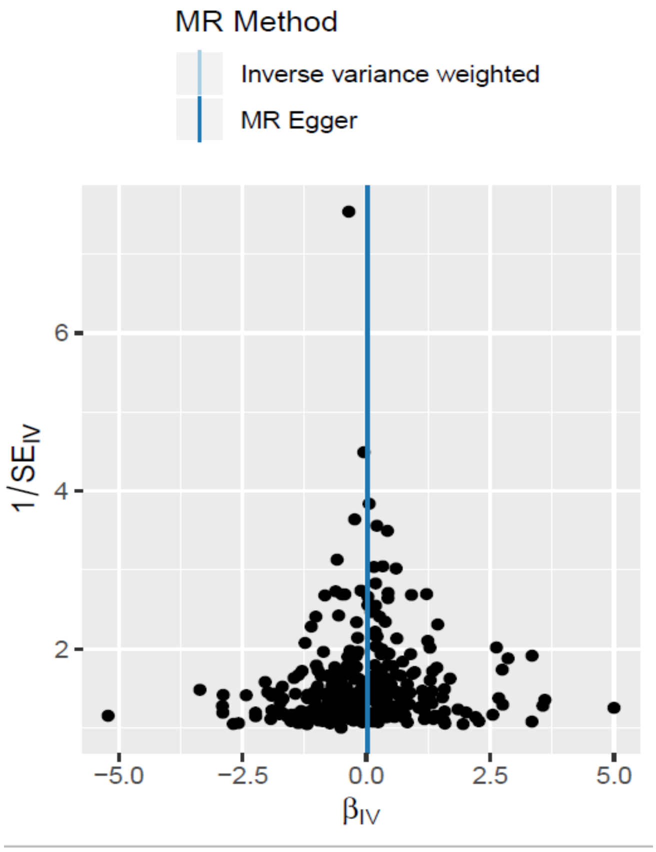

Figure S7. Leave-one-out analysis for MR with WHRadjBMI as exposure and schizophrenia as outcome

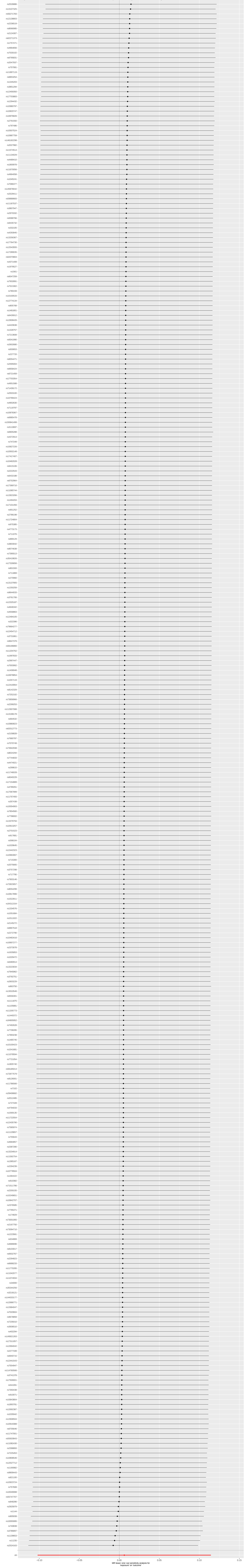

**Figure S8 Calculated power to detect a true causal effect in MR-Analysis for $WHR_{adjBMI}$ as exposure and schizophrenia as outcome**

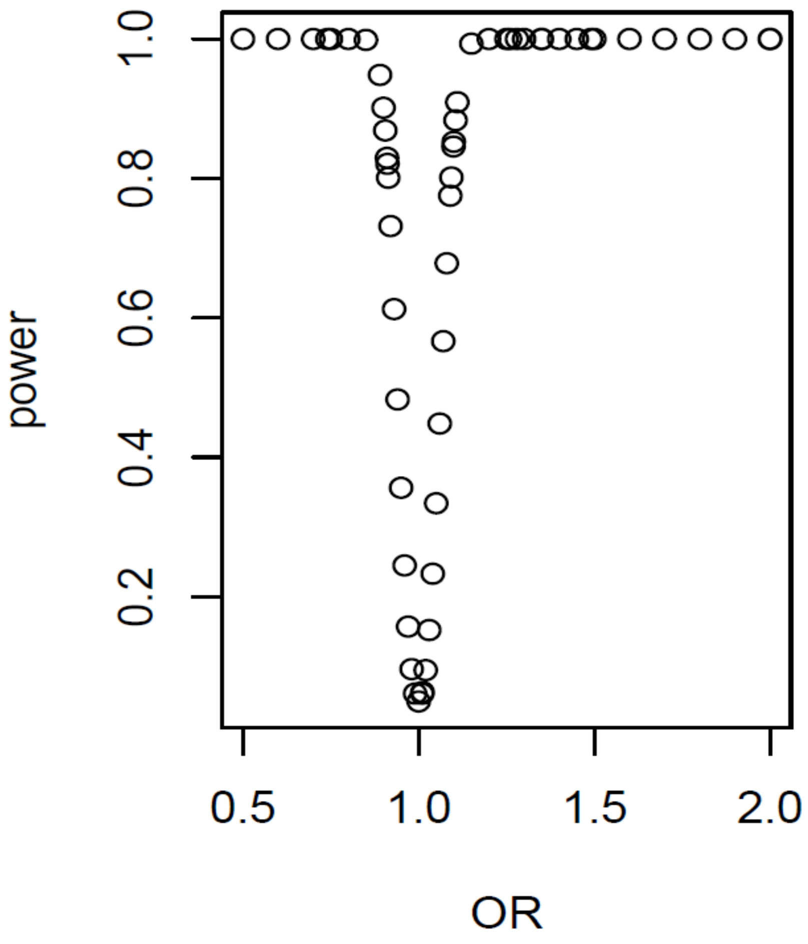

**Figure S9. Results of the single and multi SNP MR-analyses on the association between schizophrenia and $WHR_{adjBMI}$**

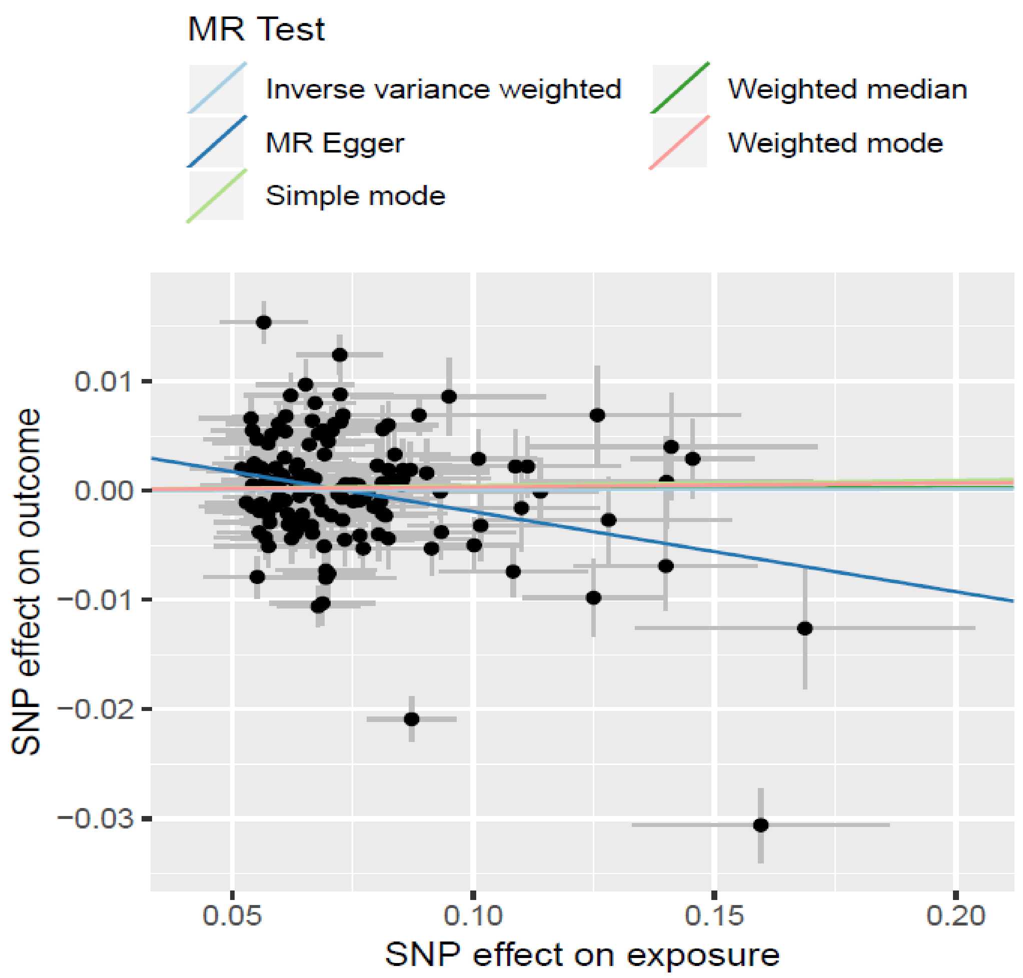


**Figure S10. Funnel plot for MR with schizophrenia as exposure and $WHR_{adjBMI}$ as outcome**

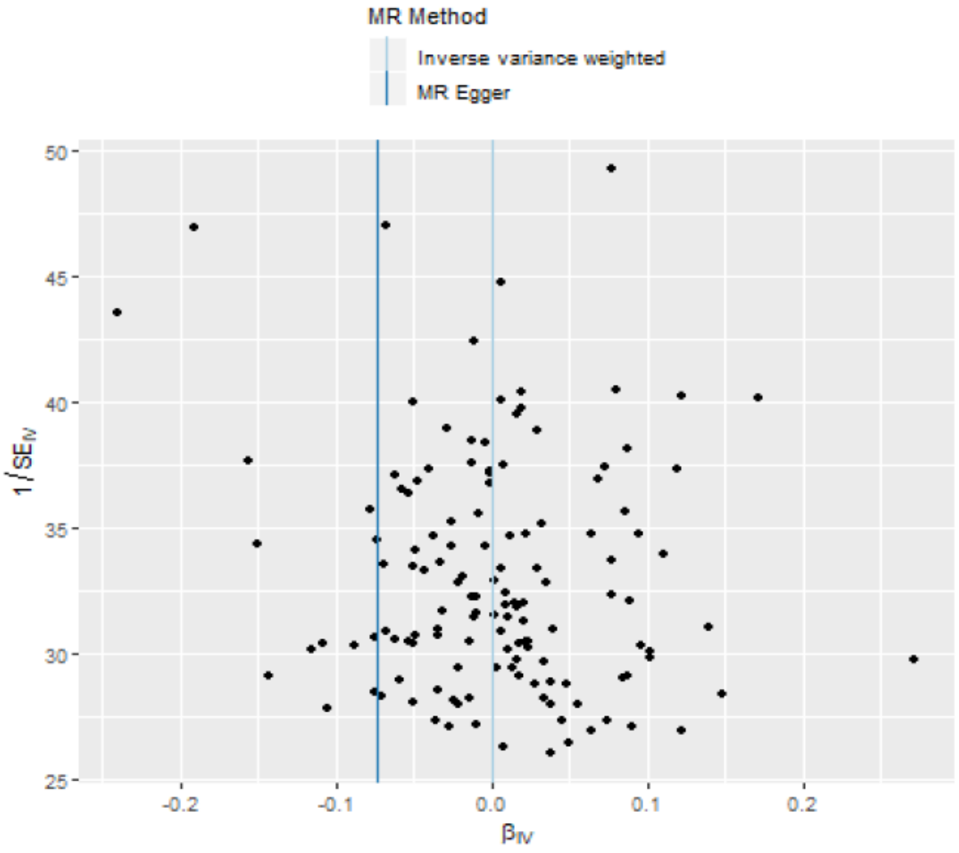

Figure S11: Results of single SNP analyses and overall estimates for causal effect of schizophrenia on WHRadjBMI

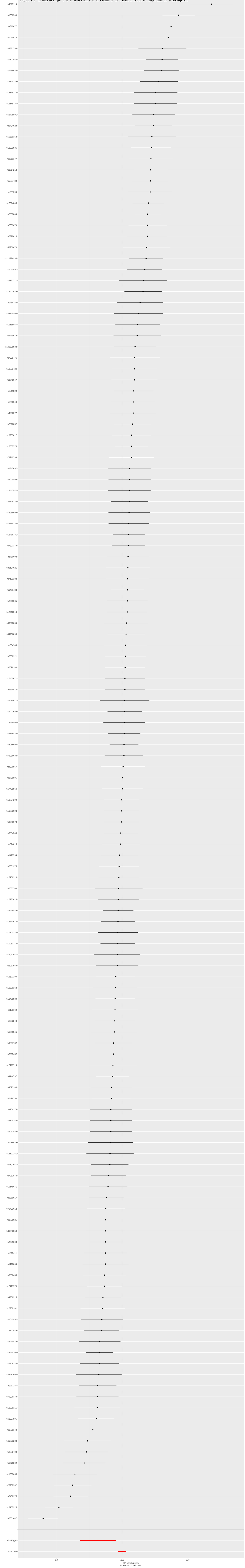

Supplement: Supplementary file 1 [file Data_Sheet_1.PDF]
